# *Helicobacter pylori* infection increases the risk of nonalcoholic fatty liver disease in diabetic population

**DOI:** 10.3389/fnut.2023.1076579

**Published:** 2023-02-01

**Authors:** Yi Chen, Ningning You, Chuchen Shen, Juju Wu, Jinshun Zhang

**Affiliations:** ^1^Department of Gastroenterology, Taizhou Hospital of Zhejiang Province Affiliated to Wenzhou Medical University, Taizhou, China; ^2^Health Management Center, Taizhou Hospital of Zhejiang Province Affiliated to Wenzhou Medical University, Taizhou, China

**Keywords:** *Helicobacter pylori*, nonalcoholic fatty liver disease, diabetes, threshold effect, glycosylated hemoglobin

## Abstract

**Background:**

The effect of *Helicobacter pylori* (*H. pylori*) on nonalcoholic fatty liver disease (NAFLD) in the population is still controversial. Diabetes and NAFLD are both metabolically related diseases, and no studies have classified the population to study the effect of *H. pylori* infection on NAFLD in diabetics.

**Methods:**

A population of people who were examined in the Taizhou Hospital Health Examination Center from 2017 to 2022 was included, and hematological indicators, body parameters, ultrasound data, and *H. pylori* detection by urea nitrogen test were collected from patients. All physical examination populations were divided into diabetic and non-diabetic populations.

**Results:**

After multivariate logistic regression, *H. pylori* infection remained an independent risk factor for NAFLD in diabetics, but it had no significant effect on NAFLD in non-diabetic population. Additionally, there was a nonlinear relationship between glycosylated hemoglobin and *H. pylori* infection in diabetic population. Moreover, the incidence of NAFLD in diabetics increased with persistent *H. pylori* infection.

**Conclusion:**

In the diabetic population, *H. pylori* infection does increase the risk of developing NAFLD. Glycemic control and eradication of *H. pylori* infection may have positive implications for reducing the incidence of NAFLD in diabetic population.

## 1. Introduction

*Helicobacter pylori* (*H. pylori*) is a Gram-negative spirochete pathogen that infects approximately half of adults population (1). The prevalence rate of *H. pylori* infection varies widely across the globe, with infection rates in Asia ranging from approximately 50–65% (2). As one of the most common infections in humans, *H. pylori* plays a major role in the development and progression of peptic ulcers, gastric mucosa-associated lymphomas and gastric cancers (3). Increasing evidence shows that *H. pylori* infection is linked to many extragastric diseases, such as liver disease, hematologic, respirator, cardiovascular and metabolism-related diseases (4, 5).

Non-alcoholic fatty liver disease (NAFLD) is becoming a leading cause of chronic liver diseases, with a 25–45% global prevalence, and is characterized by a wide range of liver conditions from steatosis to non-alcoholic steatohepatitis (NASH), cirrhosis and liver cancer. NAFLD has long been considered a manifestation of metabolic syndrome in the liver, but now there is growing evidence linking it to obesity, diabetes and insulin resistance (6–9).

Nowadays, the scientific community is increasingly interested in the relationship between NAFLD and *H. pylori* in humans (4, 10). However, it is controversial whether *H. pylori* infection contributes to the increased risk of NAFLD. Some studies suggest that *H. pylori* infection may have an impact on the development of NAFLD, possibly by increasing insulin resistance (IR), inflammatory mediator release and affecting lipid metabolism, while the eradication of *H. pylori* may play a role in reducing the risk of NAFLD (11–16). In contrast, in recent studies, many people have raised different opinions, there were two cross-sectional studies incorporating 21,456 and 71,633 healthy Chinese population found that *H. pylori* infection was not listed as a risk factor for NAFLD, besides, another observational study found no association between *H. pylori* infection and diagnosis of NAFLD in a Central European cohort (17–20). The reason for the different conclusions may be the lack of classification of the population. Some literatures have mentioned that *H. pylori* contributes to the pathogenesis of IR, a condition is closely related to NAFLD and diabetes (15, 21). In addition, diabetes and NAFLD are both metabolic syndromes, and they affect each other (22–25).

In this study, we conducted a cross-sectional survey and cohort investigation to examine whether *H. pylori* infection contributes to NAFLD risk in the diabetic population in China. Thus, we further examined the effect of *H. pylori* infection on NAFLD in the diabetic setting and explored the association between *H. pylori* infection, diabetes mellitus and NAFLD.

## 2. Materials and methods

### 2.1. Study population

The participants in this study were the people who went to Taizhou Hospital Health Examination Center for physical examination from June 2017 to June 2022. Patients with complete clinical information, such as age, gender, laboratory indices, and blood pressure, were required for the study population. All people underwent a urea nitrogen breath test, as well as liver ultrasound. Laboratory indicators include total cholesterol (TC), triglyceride (TG), low-density lipoprotein (LDL), high-density lipoprotein (HDL), alanine aminotransferase (ALT), aspartate transaminase (AST), gamma-glutamyl transpeptidase (GGT), glycated hemoglobin A1c (HbA1c) and fasting blood glucose (FBG). People with viral hepatitis, alcoholic liver disease, autoimmune liver disease, or with thyroid function problems, history of malignancy and incomplete clinical information were excluded. In total, 52,032 people were included in the study for analysis. People with diabetes mellitus who have been treated with oral medicine, FBG ≥ 7.0 mmol/l, and HbA1c ≥ 6.5% were defined as diabetic population, including 7,190 patients with diabetes. In addition, 778 of these diabetic patients who underwent multiple physical examinations (interval between physical examinations > 6 months) were categorized as *H. pylori* persistent negative, persistent infection, eradicated infection, and new infection according to their *H. pylori* infection status at the first and last physical examinations, respectively. This study was approved by the Ethics Committee of Taizhou Hospital (K20220790).

### 2.2. Collection of clinical indicators

The Health Management Center nurses measured and recorded the age, gender and blood pressure of the population. The venous blood of the patients was collected in the morning on an empty stomach for laboratory testing. Laboratory indicators, including TG, TC, HDL, LDL, ALT, AST, GGT, and FBG, were determined by automatic biochemical analyzer. The patient’s HbA1c level was measured by the glycosylated hemoglobin analyzer.

### 2.3. Detection of *Helicobacter pylori*

*Helicobacter pylori* was examined for physical examination population through ^13^C or ^14^C urease test. The process of ^13^C breath test is performed as follows: (a) collect a breath sample after 3 h of fasting; (b) take ^13^C urea capsule with warm water; (c) wait for 30 min; (d) blow to the special breath card to take the breath sample after taking the medicine; (e) analyze two samples on the instrument. The process of ^14^C breath test is as follows: (a) take ^14^C urea capsule and add water to take it; (b) Wait for 20 min; (c) exhale to the gas gathering card for 1–3 min; (d) insert the gas gathering card into the detector for detection.

### 2.4. Definition of NAFLD

Nonalcoholic fatty liver disease was defined as hepatic steatosis diagnosed by imaging or histology after excluding viral hepatitis, excessive drinking, autoimmune liver disease and hereditary liver disease (26). Ultrasonography was performed by at least two experienced ultrasound doctors who were unaware of the purpose of the study. The patient was positioned supine with the abdomen fully exposed, and the patient’s abdominal organs were examined by the ultrasound probe. The diagnostic criteria of hepatic steatosis under ultrasound were: diffuse enhancement of near-field echoes in the liver (“bright liver”) with stronger echoes than in the kidneys; indistinct intrahepatic ductal structures; and gradual attenuation of far-field echoes in the liver (13). Those who have 2 of the above 3 abdominal ultrasound findings can be diagnosed as fatty liver.

### 2.5. Definition of hypertension and dyslipidemia

Hypertension was defined as a systolic blood pressure ≥ 140 mmHg or diastolic blood pressure ≥ 90 mmHg. TC [≥ 240 mg/dl (6.20 mmol/l)], TG [>200 mg/dl (2.25 mmol/l)], HDL [<40 mg/dl (1.03 mmol/l)], LDL [>160 mg/dl (4.13 mmol/l)], meeting one of the above were defined as dyslipidemia (27).

### 2.6. Statistical analysis

In this study, continuous variables were expressed as mean ± standard deviation (SD) using *t*-test. Categorical variables were expressed as counts and percentages using chi-square test. Multivariate logistic regression was used to analyze the relationship between *Helicobacter pylori* infection and NAFLD after adjusting for confounding factors, and the odds ratio (OR) and 95% confidence interval (CI) were calculated. The generalized additive model (GAM) was used to investigate the potential nonlinear relationship between HbA1c and *H. pylori* infection. All statistical analyses were conducted using R (4.1.3) software, and *p* < 0.05 were considered statistically significant.

## 3. Result

### 3.1. Baseline characteristics

Baseline characteristics of all physical examination populations were shown in Table 1. Among the 52,032 participants, 19,755 (38.0%) were female and 32,277 (62.0%) were male. Men had a higher rate of *H. pylori* infection, 63.0% vs. 61.4%. People with diabetes had a higher rate of *H. pylori* infection, 14.4% vs. 13.5%, as well as higher *H. pylori* infection among people with NAFLD, 21.6% vs. 20.6%.

**Table 1:** Baseline characteristics of all physical examination populations.

Variables	*Helicobacter pylori*-negative (*n* = 32,323)	*Helicobacter pylori*-positive (*n* = 19,709)	Value of *p*
Gender (*n*, %)			<0.001
Female	12,470 (38.6)	7,285 (37.0)	
Male	19,853 (61.4)	12,424 (63.0)	
Age (year)	49.15 ± 12.40	49.69 ± 12.35	<0.001
Triglycerides (mmol/L)	1.85 ± 1.61	1.92 ± 1.67	<0.001
Total cholesterol (mmol/L)	5.11 ± 1.00	5.10 ± 0.99	0.039
High density lipoprotein (mmol/L)	1.42 ± 0.32	1.40 ± 0.31	<0.001
Low density lipoprotein (mmol/L)	2.79 ± 0.76	2.76 ± 0.74	<0.001
Alanine aminotransferase (U/L)	25.64 ± 19.49	25.53 ± 19.59	0.536
Aspartate transaminase (U/L)	24.30 ± 10.04	24.07 ± 10.07	0.012
Gamma-glutamyl transpeptidase (U/L)	32.65 ± 25.01	33.29 ± 25.70	0.005
Diabetes (n, %)			0.002
No	27,972 (86.5)	16,870 (85.6)	
Yes	4,351 (13.5)	2,839 (14.4)	
Diastolic blood pressure (mmHg)	76.28 ± 11.75	76.80 ± 12.03	<0.001
Systolic blood pressure (mmHg)	127.32 ± 17.82	128.22 ± 18.63	<0.001
Fasting blood glucose (mmol/L)	5.51 ± 1.49	5.56 ± 1.64	0.001
Glycated hemoglobin A1c (%)	5.89 ± 0.91	5.94 ± 1.00	<0.001
NAFLD (n, %)			0.005
No	25,670 (79.4)	15,449 (78.4)	
Yes	6,653 (20.6)	4,260 (21.6)	

### 3.2. Risk factors for NAFLD In diabetic and non-diabetic populations

**Figure 1:**
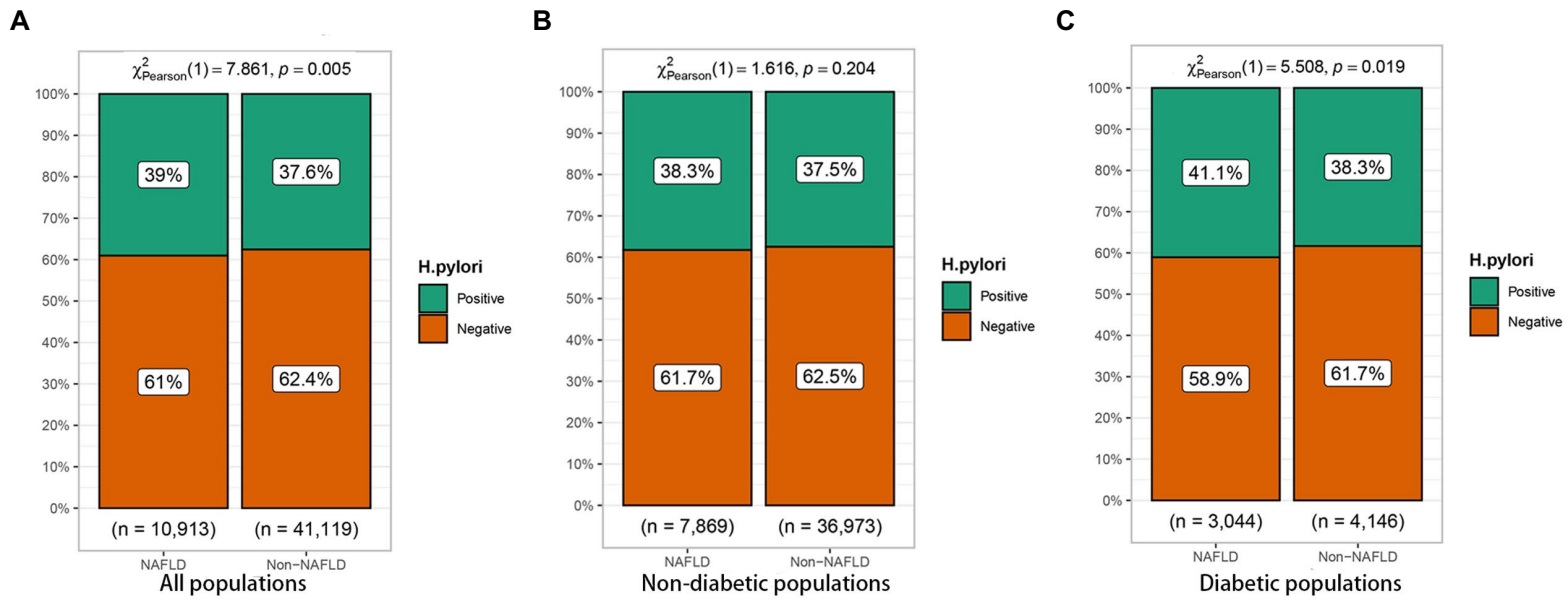
Relationship between *Helicobacter pylori* infection and nonalcoholic fatty liver disease (NAFLD) in different populations. **(A)** All populations. **(B)** Non-diabetic populations. **(C)** Diabetic populations.

To further investigate the relationship of *H. pylori* infection on the development of NAFLD in different populations, a subgroup analysis of risk factors for NAFLD was carried out in diabetic and non-diabetic populations using univariate analysis. The baseline characteristics of NAFLD population in the non-diabetic group were shown in Table 2. In the NAFLD population, the proportion of male patients increased significantly, 79.5% vs. 56.5%. However, no significant correlation was seen between *H. pylori* infection and NAFLD (*p* = 0.205). Table 3 showed the baseline characteristics of NAFLD in the diabetic population. Among patients with NAFLD, there were statistical differences between gender, age, lipids, blood liver enzymes, blood glucose, blood pressure, and *H. pylori* infection versus non-NAFLD patients. The differences in *H. pylori* infection among different populations in people with NAFLD were shown in Figure 1.

**Table 2:** The baseline characteristics of NAFLD population in the non-diabetic group.

Variables	Non-NAFLD (*n* = 36,973)	NAFLD (*n* = 7,869)	Value of *p*
Gender (*n*, %)			<0.001
Female	16,100 (43.5)	1,617 (20.5)	
Male	20,873 (56.5)	6,252 (79.5)	
Age (year)	48.25 ± 12.31	47.38 ± 11.25	<0.001
Triglycerides (mmol/L)	1.58 ± 1.18	2.74 ± 2.06	<0.001
Total cholesterol (mmol/L)	5.05 ± 0.97	5.26 ± 0.98	<0.001
High density lipoprotein (mmol/L)	1.47 ± 0.32	1.23 ± 0.26	<0.001
Low density lipoprotein (mmol/L)	2.73 ± 0.73	2.94 ± 0.75	<0.001
Alanine aminotransferase (U/L)	21.59 ± 14.64	40.26 ± 27.11	<0.001
Aspartate transaminase (U/L)	22.92 ± 8.50	28.77 ± 12.10	<0.001
Gamma-glutamyl transpeptidase (U/L)	28.52 ± 22.37	46.57 ± 28.74	<0.001
Diastolic blood pressure (mmHg)	74.88 ± 11.59	80.77 ± 11.50	<0.001
Systolic blood pressure (mmHg)	124.97 ± 17.53	131.91 ± 16.63	<0.001
Fasting blood glucose (mmol/L)	5.06 ± 0.53	5.31 ± 0.58	<0.001
Glycated hemoglobin A1c (%)	5.60 ± 0.34	5.76 ± 0.34	<0.001
H. pylori			0.205
negative	23,113 (62.5)	4,859 (61.7)	
positive	12,860 (37.5)	3,010 (38.3)	

**Table 3:** Baseline characteristics of the NAFLD population in the diabetic group.

Variables	Non-NAFLD (*n* = 4,146)	NAFLD (*n* = 3,044)	Value of *p*
Gender (n, %)			<0.001
Female	1,267 (30.6)	771 (25.3)	
Male	2,879 (69.4)	2,273 (74.7)	
Age (year)	59.18 ± 10.87	54.41 ± 10.58	<0.001
Triglycerides (mmol/L)	1.97 ± 1.70	3.15 ± 3.00	<0.001
Total cholesterol (mmol/L)	5.14 ± 1.10	5.38 ± 1.16	<0.001
High density lipoprotein (mmol/L)	1.40 ± 0.31	1.25 ± 0.26	<0.001
Low density lipoprotein (mmol/L)	2.79 ± 0.82	2.93 ± 0.81	<0.001
Alanine aminotransferase (U/L)	23.78 ± 16.00	39.65 ± 27.18	<0.001
Aspartate transaminase (U/L)	23.06 ± 9.77	29.87 ± 15.48	<0.001
Gamma-glutamyl transpeptidase (U/L)	34.11 ± 24.80	49.73 ± 29.75	<0.001
Diastolic blood pressure (mmHg)	78.29 ± 11.41	82.26 ± 11.75	<0.001
Systolic blood pressure (mmHg)	136.15 ± 19.33	137.78 ± 18.16	<0.001
Fasting blood glucose (mmol/L)	8.05 ± 2.81	8.30 ± 2.61	<0.001
Glycated hemoglobin A1c (%)	7.58 ± 1.54	7.71 ± 1.45	<0.001
*H. pylori*			0.019
negative	2,557 (61.7)	1794 (58.9)	
positive	1,589 (38.3)	1,250 (41.1)	

### 3.3. Effect of *Helicobacter pylori* infection on NAFLD

**Figure 2:**
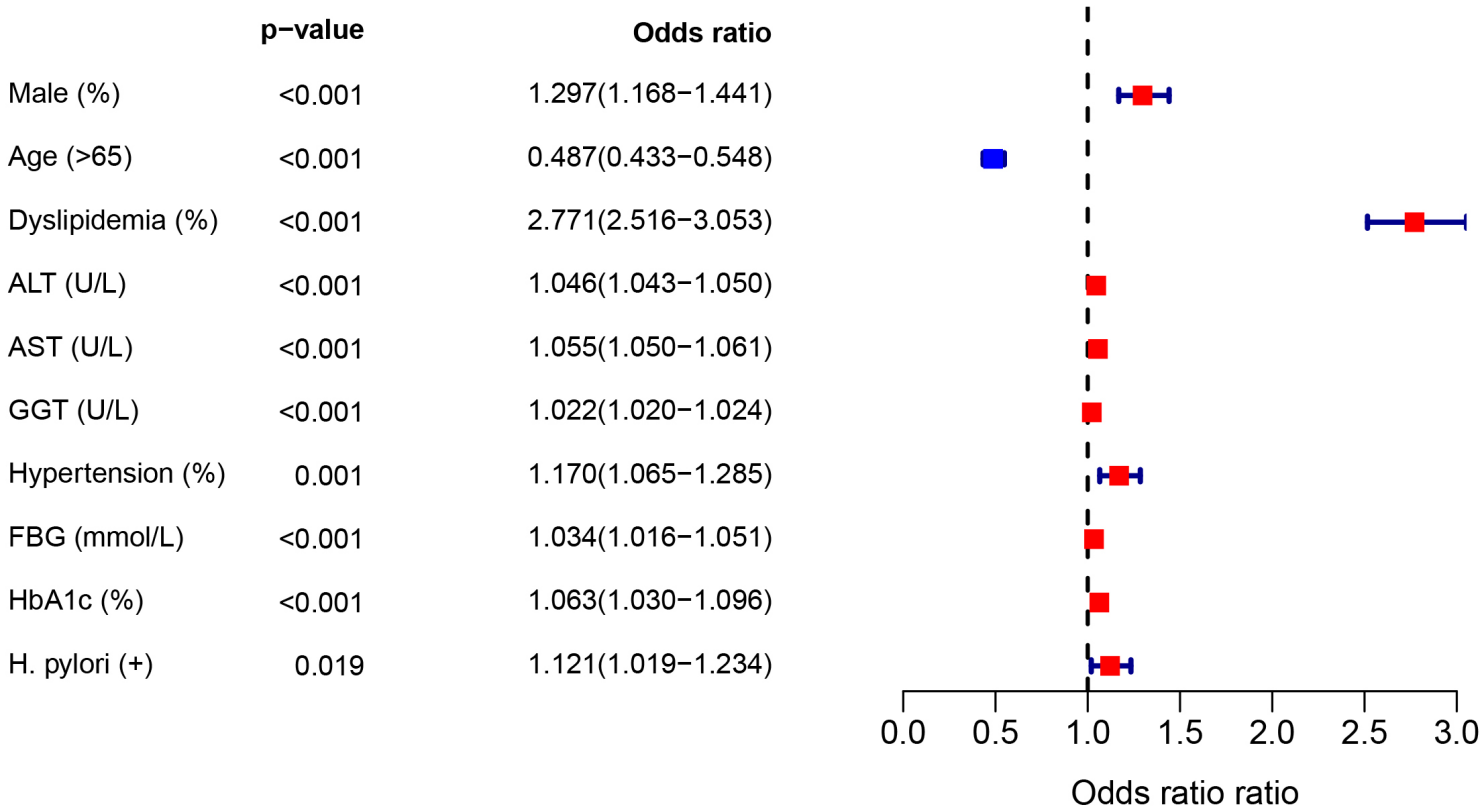
Univariate analysis of NAFLD risk factors in diabetes population.

In the diabetic population, univariate logistic regression analysis showed an increased risk of NAFLD due to *H. pylori* (OR = 1.12, 95%CI: 1.02–1.23), as shown in Figure 2. In addition, gender, age, dyslipidemia, blood liver enzymes, hypertension, and FBG were all risk factors for NAFLD (*p* < 0.05). In order to control the influence of confounding factors, in addition to gender and age, dyslipidemia, hypertension, FBG and blood liver enzymes were adjusted, respectively. *Helicobacter pylori* infection was still a risk factor for NAFLD, Table 4.

**Table 4:** Relationship between *H. pylori* infection and NAFLD in different regression models.

	OR (95%CI)	Value of *p*
Model 1	1.13 (1.03–1.25)	0.013
Model 2	1.11 (1.00–1.22)	0.046
Model 3	1.12 (1.02–1.24)	0.018
Model 4	1.12 (1.02–1.24)	0.019
Model 5	1.14 (1.03–1.27)	0.015
Model 1 is adjusted for age, sex.		
Model 2 is adjusted for age, sex, dyslipidemias.		
Model 3 is adjusted for age, sex, hypertension.		
Model 4 is adjusted for age, sex, fasting blood glucose.		
Model 5 is adjusted for age, sex, blood liver enzymes.		

### 3.4. Effect of HbA1c on *Helicobacter pylori* infection

**Figure 3:**
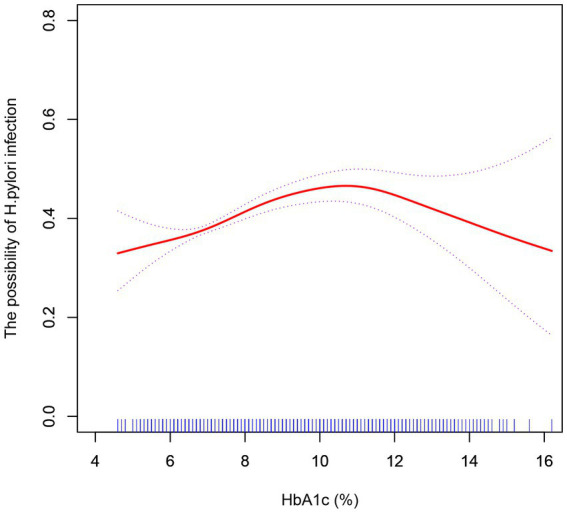
After adjustment for confounding factors (age, sex), a smoothed curve fit was used to show a non-linear correlation between HbA1c and *H. pylori* infection.

We further explored the effect of HbA1c on *H. pylori* infection in diabetic patients. In curve fitting, we found a nonlinear relationship between HbA1c and *H. pylori* infection, Figure 3. After adjusting the sex and age, the impact of HbA1c on *H. pylori* infection was analyzed by using two-stage linear regression, Table 5. When HbA1c did not reach the threshold of 10.8%, the risk of *H. pylori* infection increased with elevated HbA1c (OR = 1.13, *p* < 0.001), but after reaching the threshold, the HP infection rate decreased with the increase of HbA1c (OR = 0.83, *p* = 0.013).

**Table 5:** No-linear relationship between *H. pylori* infection and HbA1c.

	OR (95%CI)	Value of *p*
Binary logical regression	1.08 (1.05–1.12)	<0.001
Piecewise logistic regression		
HbA1c < 10.8	1.13 (1.09–1.18)	<0.001
HbA1C ≥ 10.8	0.83 (0.71–0.97)	0.013

### 3.5. Association between *Helicobacter pylori* infection status and NAFLD

**Figure 4:**
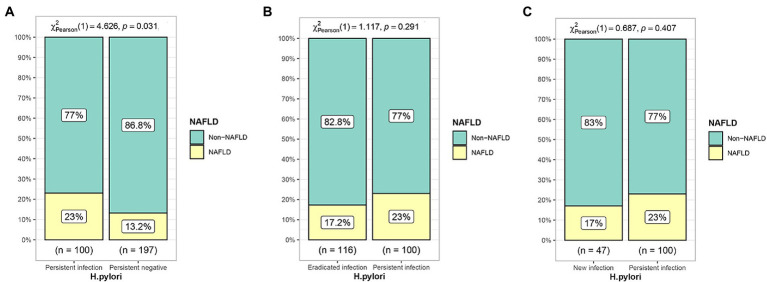
Effect of different *H. pylori* infection status on the development of NAFLD. **(A)** Persistent infection group compared with persistent negative group. **(B)** Eradicated infection group compared with persistent infection group. **(C)** New infection group compared with persistent infection group.

Of the 778 diabetic patients, 460 (59.1%) patients did not have NAFLD on first physical examination. The incidence of NAFLD was 13.2% in the persistent negative group, 23.0% in the persistent positive group, 17.2% in the eradicated infection group, and 17.0% in the new infection group. The incidence of NAFLD was significantly lower in the persistent negative group compared to the persistent infection group (*p* = 0.031); the incidence of NAFLD in the new infection and eradicated infection groups was significantly lower compared with the persistent positive group, but non-statistically significant difference was found (*p* > 0.05), as shown in Figure 4.

## 4. Discussion

Nonalcoholic fatty liver disease is a metabolic disease that can manifest as simple fatty liver in its early stages, but as the disease progresses, it can manifest as steatohepatitis, cirrhosis, and even liver cancer (28). The pathogenesis of NAFLD may be related to genetic and environmental, as well as metabolic factors, and some studies have suggested an association between NAFLD and *H. pylori* infection in recent years (29, 30). Several studies have found that *H. pylori* upregulates the expression of various inflammatory factors, including C-reactive protein, tumor necrosis factor, various interleukins, and promotes insulin resistance levels (10, 31). In addition, *H. pylori* infection may increase intestinal permeability, cause intestinal microbial disorders, and promote the entry of bacterial toxins into the liver, thus leading to chronic inflammation of liver (12, 32, 33).

However, the relationship between NAFLD and *H. pylori* infection remains controversial. In addition, there are no studies that classify populations according to the presence or absence of diabetes to exclude effect of diabetes on NAFLD in *H. pylori* infection status. Our study, in a large sample population, confirmed the relationship between NAFLD and *H. pylori* infection. However, due to the fact that conditions such as insulin resistance and genetic susceptibility in the diabetic population differ from those in the non-diabetic population, there may be differences in the role of *H. pylori* infection on NAFLD, so we studied diabetic patients separately. In the non-diabetic population, *H. pylori* infection did not significantly contribute to NAFLD. However, we found a statistically significant association between *H. pylori* infection and NAFLD in the diabetic population. After adjusting for multiple confounders in multifactorial logistic regression, *H. pylori* infection remained an independent risk factor for the development of NAFLD. Moreover, we found a significant relationship between *H. pylori* infection and HbA1c in diabetic patients, with or without adjustment for confounding factors, which was consistent with previous studies (34, 35). However, there was a threshold effect between HbA1c and *H. pylori* infection, which may not be mentioned in other studies. At HbA1c < 10.8%, the risk of *H. pylori* infection increased with increasing HbA1c. However, when HbA1c > 10.8%, the rate of *H. pylori* infection decreased instead. This could mean that prolonged poor glycemic control leads to changes in the gastrointestinal flora, as well as the intestinal mucosal barrier, and that other intestinal microorganisms are more likely to cause infection than *H. pylori* (36, 37). Therefore, the control of blood glucose in diabetic patients is still of positive significance for reducing *H. pylori* infection. However, this still needs to be validated in depth by prospective cohort studies. Furthermore, as confirmed by our cohort study, persistent *H. pylori* infection significantly increases the incidence of NAFLD. Eradicating *H. pylori* infection in diabetic population may have positive implications for reducing the incidence of NAFLD.

Previously, lifestyle changes and diet control were the main treatment options for NAFLD. For the past few years, a variety of drugs such as anti-diabetic drugs, anti-obesity drugs, and antioxidants have been investigated for the treatment of NAFLD as well as NASH (38, 39). These drugs focus on regulating metabolic dysregulation; however, the complex pathogenesis of NAFLD and the important role of immune-mediated inflammation in NAFLD development may lead to the failure of many drug candidates to achieve the expected efficacy at the late stage of the trial (38, 40). Therefore, testing for *H. pylori* infection and treating it before treating NAFLD may have some positive implications for subsequent drug therapy.

Our study confirms that *H. pylori* infection plays an independent role in the development of NAFLD in diabetic population. This may be the first time that the effect of *H. pylori* infection on NAFLD has been studied in a large diabetic population. In addition, we explore the nonlinear relationship of HbA1c to *H. pylori* infection in a diabetic population. The survey, however, still has several limitations. First of all, this is a single-center study with its own limitations. Multicenter, longitudinal studies may be needed to provide more robust evidence. Second, the diagnosis of NAFLD is mainly based on ultrasonography and lacks confirmation by liver biopsy data (41). However, due to the high sensitivity and specificity of ultrasonography for the diagnosis of steatohepatitis, this test is widely used in clinical practice (42). In addition, the invasive nature of liver puncture makes it inappropriate for such a large sample of people undergoing physical examination. Finally, although some methods are being used to adjust for confounding factors, there may still be a potential influence of other factors.

## 5. Conclusion

*Helicobacter pylori* infection is an independent risk factor for NAFLD in diabetic population. In addition, long-term *H. pylori* infection may increase the incidence of NAFLD. Controlling glycemia and eradicating *H. pylori* infection in diabetic population may benefit to reduce the incidence of NAFLD.

## 6. Data availability statement

The raw data supporting the conclusions of this article will be made available by the authors, without undue reservation.

## Ethics statement

The studies involving human participants were reviewed and approved by the Ethics Committee of Taizhou Hospital (K20220790). The patients/participants provided their written informed consent to participate in this study.

## Author contributions

JZ made great contributions to the concept, design and data acquisition of the article. YC and NY was mainly involved in data analysis. CS and JW were involved in article writing. All authors contributed to the article and approved the submitted version.

## Funding

This study was supported by the Taizhou Science and Technology Program (No. 1901ky18).

## Conflict of interest

The authors declare that the research was conducted in the absence of any commercial or financial relationships that could be construed as a potential conflict of interest.

## Publisher’s note

All claims expressed in this article are solely those of the authors and do not necessarily represent those of their affiliated organizations, or those of the publisher, the editors and the reviewers. Any product that may be evaluated in this article, or claim that may be made by its manufacturer, is not guaranteed or endorsed by the publisher.

## References

[1] Wotherspoon AC Ortiz-Hidalgo C Falzon MR Isaacson PG. *Helicobacter pylori*-associated gastritis and primary B-cell gastric lymphoma. Lancet. (1991) 338:1175–6. doi: 10.1016/0140-6736(91)92035-z, PMID: 1682595

[2] Zamani M Ebrahimtabar F Zamani V Miller WH Alizadeh-Navaei R Shokri-Shirvani J. Systematic review with meta-analysis: the worldwide prevalence of *Helicobacter pylori* infection. Aliment Pharmacol Ther. (2018) 47:868–76. doi: 10.1111/apt.14561, PMID: 29430669

[3] Vakil N Malfertheiner P Chey WD. *Helicobacter pylori* infection. N Engl J Med. (2010) 363:595–6. doi: 10.1056/NEJMc1006158 20818897

[4] de Korwin JD Ianiro G Gibiino G Gasbarrini A. *Helicobacter pylori* infection and extragastric diseases in 2017. Helicobacter. (2017) 22:1. doi: 10.1111/hel.12411, PMID: 28891133

[5] Pellicano R Ianiro G Fagoonee S Settanni CR Gasbarrini A. Review: extragastric diseases and *Helicobacter pylori*. Helicobacter. (2020) 25:e12741. doi: 10.1111/hel.12741 32918343

[6] Lonardo A Bellentani S Argo CK Ballestri S Byrne CD Caldwell SH. Epidemiological modifiers of non-alcoholic fatty liver disease: focus on high-risk groups. Dig Liver Dis. (2015) 47:997–1006. doi: 10.1016/j.dld.2015.08.004, PMID: 26454786

[7] Younossi Z Anstee QM Marietti M Hardy T Henry L Eslam M. Global burden of NAFLD and NASH: trends, predictions, risk factors and prevention. Nat Rev Gastroenterol Hepatol. (2018) 15:11–20. doi: 10.1038/nrgastro.2017.109, PMID: 28930295

[8] Fazel Y Koenig AB Sayiner M Goodman ZD Younossi ZM. Epidemiology and natural history of non-alcoholic fatty liver disease. Metabolism. (2016) 65:1017–25. doi: 10.1016/j.metabol.2016.01.012 26997539

[9] Fabbrini E Magkos F. Hepatic Steatosis as a marker of metabolic dysfunction. Nutrients. (2015) 7:4995–5019. doi: 10.3390/nu7064995, PMID: 26102213 PMC4488828

[10] Cheng DD He C Ai HH Huang Y Lu NH. The possible role of helicobacter pylori infection in non-alcoholic fatty liver disease. Front Microbiol. (2017) 8:743. doi: 10.3389/fmicb.2017.00743, PMID: 28539915 PMC5423951

[11] Polyzos SA Kountouras J Papatheodorou A Patsiaoura K Katsiki E Zafeiriadou E. Helicobacter pylori infection in patients with nonalcoholic fatty liver disease. Metabolism. (2013) 62:121–6. doi: 10.1016/j.metabol.2012.06.007 22841522

[12] Sumida Y Kanemasa K Imai S Mori K Tanaka S Shimokobe H. Helicobacter pylori infection might have a potential role in hepatocyte ballooning in nonalcoholic fatty liver disease. J Gastroenterol. (2015) 50:996–1004. doi: 10.1007/s00535-015-1039-2, PMID: 25622927

[13] Chen CX Mao YS Foster P Zhu ZW Du J Guo CY. Possible association between *Helicobacter pylori* infection and nonalcoholic fatty liver disease. Appl Physiol Nutr Metab. (2017) 42:295–301. doi: 10.1139/apnm-2016-0499 28177748

[14] Lu LJ Hao NB Liu JJ Li X Wang RL. Correlation between *Helicobacter pylori* infection and metabolic abnormality in general population: a cross-sectional study. Gastroenterol Res Pract. (2018) 2018:7410801. doi: 10.1155/2018/7410801, PMID: 29743888 PMC5883933

[15] Abdel-Razik A Mousa N Shabana W Refaey M Elhelaly R Elzehery R. Helicobacter pylori and non-alcoholic fatty liver disease: a new enigma? Helicobacter. (2018) 23:e12537. doi: 10.1111/hel.12537, PMID: 30246507

[16] Kim TJ Sinn DH Min YW Son HJ Kim JJ Chang Y. A cohort study on helicobacter pylori infection associated with nonalcoholic fatty liver disease. J Gastroenterol. (2017) 52:1201–10. doi: 10.1007/s00535-017-1337-y, PMID: 28382402

[17] Wang W Fan M Gong R Zhang Y Zeng J Xu S. *Helicobacter pylori* infection is not an independent risk factor of non-alcoholic fatty liver disease in China. BMC Gastroenterol. (2022) 22:81. doi: 10.1186/s12876-022-02148-6, PMID: 35209867 PMC8867781

[18] Fan N Peng L Xia Z Zhang L Wang Y Peng Y. *Helicobacter pylori* infection is not associated with non-alcoholic fatty liver disease: a cross-sectional study in China. Front Microbiol. (2018) 9:73. doi: 10.3389/fmicb.2018.00073, PMID: 29445363 PMC5797778

[19] Cai O Huang Z Li M Zhang C Xi F Tan S. Association between helicobacter pylori infection and nonalcoholic fatty liver disease: a single-center clinical study. Gastroenterol Res Pract. (2018) 2018:8040262. doi: 10.1155/2018/8040262, PMID: 29527224 PMC5828541

[20] Wernly S Wernly B Semmler G Völkerer A Rezar R Semmler L. Non-alcoholic fatty liver disease is not independently associated with *Helicobacter pylori* in a central European screening cohort. Minerva Med. (2022). doi: 10.23736/s0026-4806.22.07928-9 35384436

[21] Azami M Baradaran HR Dehghanbanadaki H Kohnepoushi P Saed L Moradkhani A. Association of Helicobacter pylori infection with the risk of metabolic syndrome and insulin resistance: an updated systematic review and meta-analysis. Diabetol Metab Syndr. (2021) 13:145. doi: 10.1186/s13098-021-00765-x, PMID: 34922625 PMC8684139

[22] Mantovani A Petracca G Beatrice G Tilg H Byrne CD Targher G. Non-alcoholic fatty liver disease and risk of incident diabetes mellitus: an updated meta-analysis of 501 022 adult individuals. Gut. (2021) 70:962–9. doi: 10.1136/gutjnl-2020-322572, PMID: 32938692

[23] Chang Y Jung HS Yun KE Cho J Cho YK Ryu S. Cohort study of non-alcoholic fatty liver disease, NAFLD fibrosis score, and the risk of incident diabetes in a Korean population. Am J Gastroenterol. (2013) 108:1861–8. doi: 10.1038/ajg.2013.349, PMID: 24100261

[24] Adams LA Anstee QM Tilg H Targher G. Non-alcoholic fatty liver disease and its relationship with cardiovascular disease and other extrahepatic diseases. Gut. (2017) 66:1138–53. doi: 10.1136/gutjnl-2017-313884, PMID: 28314735

[25] Mantovani A Byrne CD Bonora E Targher G. Nonalcoholic fatty liver disease and risk of incident type 2 diabetes: a meta-analysis. Diabetes Care. (2018) 41:372–82. doi: 10.2337/dc17-1902 29358469

[26] Chalasani N Younossi Z Lavine JE Charlton M Cusi K Rinella M. The diagnosis and management of nonalcoholic fatty liver disease: practice guidance from the American Association for the Study of Liver Diseases. Hepatology. (2018) 67:328–57. doi: 10.1002/hep.29367, PMID: 28714183

[27] Kopin L Lowenstein C. Dyslipidemia. Ann Intern Med. (2017) 167:Itc81–itc96. doi: 10.7326/aitc201712050 29204622

[28] Ofosu A Ramai D Reddy M. Non-alcoholic fatty liver disease: controlling an emerging epidemic, challenges, and future directions. Ann Gastroenterol. (2018) 31:288–95. doi: 10.20524/aog.2018.0240, PMID: 29720854 PMC5924851

[29] Aron-Wisnewsky J Vigliotti C Witjes J Le P Holleboom AG Verheij J. Gut microbiota and human NAFLD: disentangling microbial signatures from metabolic disorders. Nat Rev Gastroenterol Hepatol. (2020) 17:279–97. doi: 10.1038/s41575-020-0269-9, PMID: 32152478

[30] Liu R Liu Q He Y Shi W Xu Q Yuan Q. Association between helicobacter pylori infection and nonalcoholic fatty liver: a meta-analysis. Medicine (Baltimore). (2019) 98:e17781. doi: 10.1097/md.0000000000017781, PMID: 31689846 PMC6946209

[31] Polyzos SA Kountouras J Zavos C Deretzi G. The association between helicobacter pylori infection and insulin resistance: a systematic review. Helicobacter. (2011) 16:79–88. doi: 10.1111/j.1523-5378.2011.00822.x, PMID: 21435084

[32] Fukuda Y Bamba H Okui M Tamura K Tanida N Satomi M. *Helicobacter pylori* infection increases mucosal permeability of the stomach and intestine. Digestion. (2001) 63:93–6. doi: 10.1159/000051918, PMID: 11173917

[33] Boursier J Mueller O Barret M Machado M Fizanne L Araujo-Perez F. The severity of nonalcoholic fatty liver disease is associated with gut dysbiosis and shift in the metabolic function of the gut microbiota. Hepatology. (2016) 63:764–75. doi: 10.1002/hep.28356, PMID: 26600078 PMC4975935

[34] Chen J Xing Y Zhao L Ma H. The association between helicobacter pylori infection and Glycated hemoglobin a in diabetes: a meta-analysis. J Diabetes Res. (2019) 2019:3705264–10. doi: 10.1155/2019/3705264, PMID: 31583248 PMC6754895

[35] Dai YN Yu WL Zhu HT Ding JX Yu CH Li YM. Is *Helicobacter pylori* infection associated with glycemic control in diabetics? World J Gastroenterol. (2015) 21:5407–16. doi: 10.3748/wjg.v21.i17.5407, PMID: 25954115 PMC4419082

[36] van Heck JIP Gacesa R Stienstra R Fu J Zhernakova A Harmsen HJM. The gut microbiome composition is altered in long-standing type 1 diabetes and associates with glycemic control and disease-related complications. Diabetes Care. (2022) 45:2084–94. doi: 10.2337/dc21-2225, PMID: 35766965

[37] Li J Zhang H Wang G. Correlations between inflammatory response, oxidative stress, intestinal pathological damage and intestinal flora variation in rats with type 2 diabetes mellitus. Eur Rev Med Pharmacol Sci. (2020) 24:10162–8. doi: 10.26355/eurrev_202010_23236, PMID: 33090424

[38] Negi CK Babica P Bajard L Bienertova-Vasku J Tarantino G. Insights into the molecular targets and emerging pharmacotherapeutic interventions for nonalcoholic fatty liver disease. Metabolism. (2022) 126:154925. doi: 10.1016/j.metabol.2021.154925, PMID: 34740573

[39] Polyzos SA Kang ES Boutari C Rhee EJ Mantzoros CS. Current and emerging pharmacological options for the treatment of nonalcoholic steatohepatitis. Metabolism. (2020) 111:154203. doi: 10.1016/j.metabol.2020.154203, PMID: 32151660

[40] Reccia I Kumar J Akladios C Virdis F Pai M Habib N. Non-alcoholic fatty liver disease: a sign of systemic disease. Metabolism. (2017) 72:94–108. doi: 10.1016/j.metabol.2017.04.011, PMID: 28641788

[41] Papatheodoridi M Cholongitas E. Diagnosis of non-alcoholic fatty liver disease (NAFLD): current concepts. Curr Pharm Des. (2018) 24:4574–86. doi: 10.2174/1381612825666190117102111 30652642

[42] Dasarathy S Dasarathy J Khiyami A Joseph R Lopez R McCullough AJ. Validity of real time ultrasound in the diagnosis of hepatic steatosis: a prospective study. J Hepatol. (2009) 51:1061–7. doi: 10.1016/j.jhep.2009.09.001, PMID: 19846234 PMC6136148

